# Effects of Modified Thermoplastic Starch on Crystallization Kinetics and Barrier Properties of PLA

**DOI:** 10.3390/polym13234125

**Published:** 2021-11-26

**Authors:** Apoorva Kulkarni, Ramani Narayan

**Affiliations:** Department of Chemical Engineering & Material Science, Michigan State University, East Lansing, MI 48824, USA; narayan@egr.msu.edu

**Keywords:** bio-based polymers, biodegradable polymers, barrier properties, packaging, PLA

## Abstract

This study reports on using reactive extrusion (REX) modified thermoplastic starch particles as a bio-based and biodegradable nucleating agent to increase the rate of crystallization, percent crystallinity and improve oxygen barrier properties while maintaining the biodegradability of PLA. Reactive blends of maleated thermoplastic starch (MTPS) and PLA were prepared using a ZSK-30 twin-screw extruder; 80% glycerol was grafted on the starch during the preparation of MTPS as determined by soxhlet extraction with acetone. The crystallinity of PLA was found to increase from 7.7% to 28.6% with 5% MTPS. The crystallization temperature of PLA reduced from 113 °C to 103 °C. Avrami analysis of the blends showed that the crystallization rate increased 98-fold and t_1/2_ was reduced drastically from 20 min to <1 min with the addition of 5% MTPS compared to neat PLA. Observation from POM confirmed that the presence of MTPS in the PLA matrix significantly increased the rate of formation and density of spherulites. Oxygen and water vapor permeabilities of the solvent-casted PLA/MTPS films were reduced by 33 and 19% respectively over neat PLA without causing any detrimental impacts on the mechanical properties (α = 0.05). The addition of MTPS to PLA did not impact the biodegradation of PLA in an aqueous environment.

## 1. Introduction

Replacing petro-fossil carbon with bio-based carbon in polymers offers a reduced material carbon footprint and managed end of life [1,2,3]. The most widely studied and commercial bioplastic is polylactide (PLA) polymer. It is manufactured commercially by NatureWorks LLC, MN, USA (https://www.natureworksllc.com/) (accessed on 2 June 2021) (150 kton plant in Blair, Nebraska), and Total Corbion (https://www.total-corbion.com/) (accessed on 22 September 2021) (a total capacity of 175 kton with plants in Thailand, and France). PLA is 100% bio-based and at its end-of-life recyclable [4] or industrially compostable. However, several property deficiencies in PLA have restricted its use in many packaging applications—primarily a low percentage of crystallinity and slow rate of crystallization. Nucleating agents like talc, nanocrystalline cellulose, hydrazine, PDLA, and other molecules have been used for increasing the crystallization rate and percent crystallinity of neat PLA [5,6,7,8,9,10,11,12].

PLA has mechanical and barrier properties comparable to polystyrene (PS) and thermal properties similar to polyethylene terephthalate (PET). The water vapor permeability of PLA films is low (1–4 × 10^−14^ kg·m^2^/s·m·Pa) [13,14,15,16] because of its hydrophobic nature. However, the oxygen permeability of PLA is very high as compared to PET, which limits its use in many packaging applications [17,18,19]. Table 1 shows a comparison of the literature values of mechanical, thermal, barrier, and tensile properties of some commonly used polymers in packaging including PLA, Polyethylene terephthalate (PET), Low density polyethylene (LDPE), polystyrene (PS), polypropylene (PP) and starch.

**Figure 1:**
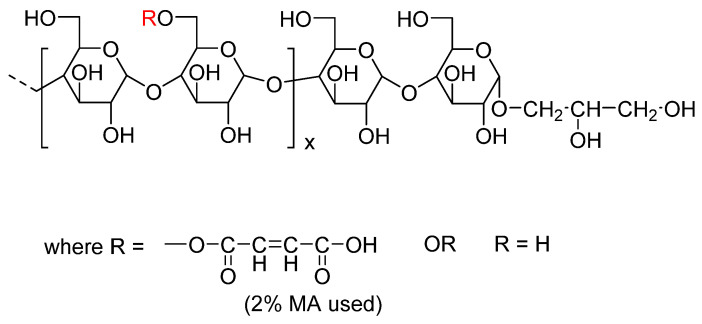
Structure of maleated thermoplastic starch (MTPS).

Starch is an abundant, inexpensive, 100% bio-based, and completely biodegradable polymer. Starch consists of two main units—(1) amylose which is a linear polymer containing chains of α-1,4-anhydroglucose units which are mainly responsible for film-forming abilities and (2) amylopectin which is a highly branched polymer containing α-1,4-anhydroglucose units and α-1,6-glycosidic branched chains [18,19,20]. The ratio of amylose and amylopectin is different in different starches [13]. The melting temperature of pure starch is above its decomposition temperature. Therefore, it does not flow on thermal processing [21]. To make starch processable, plasticizers such as water, glycerol, sorbitol are used [21,22]. However, such thermoplastic starches (TPS) have poor dimensional stability and reduced mechanical properties with time [21,22]. More problematic is the leaching of the plasticizer (glycerol for example) over time contributing to brittleness and making the film surface tacky and unusable. In our group, we have synthesized a maleated thermoplastic starch (MTPS) using reactive extrusion (REX) in which the glycerol plasticizer is covalently bonded to the starch, thereby eliminating glycerol migration and maintaining good processability [23,24]. The structure of the glycerylated starch polymer is shown below in Figure 1 and described in our earlier papers [23,24,25].

REX offers several advantages over traditional batch and flow reactors (CSTR, PFR) like fast reaction time, enhanced heat and mass transfer, better mixing and does not require any solvents [26]. Starch based films have shown some desirable properties like high barrier to oxygen and CO_2_ which is useful in packaging [27,28]. The oxygen permeability of starch films ranges between 0.4–2.5 × 10^−13^ cm^3^/m·s·Pa. Because of these advantages, different types of starch are often blended with PLA to reduce its cost and improve properties. However, pure starch and PLA blends are thermodynamically immiscible due to the hydrophobic nature of PLA and the hydrophilic nature of starch. Hence, the resulting system shows reduced strength and ductility compared to neat PLA. Several strategies have been tried to improve the compatibility by modifying either PLA or starch [29,30,31]. Studies have also shown the effect of starch and thermoplastic starch as a completely bio-based and biodegradable nucleating agent for PLA as opposed to inorganic talc [32]. Sun et al. studied the crystallization kinetics of PLA and starch composites and found that the addition of 1% of starch increased the crystallization rate considerably [33]. Jang et al. studied the thermal properties and morphology of PLA/starch blends using MA as compatibilizer and it was found that MA modified starch was much more compatible with PLA than pure starch [34]. Starch is hydrophilic and highly water sensitive. However, encapsulating the starch within the hydrophobic PLA matrix can mitigate this issue. This is in fact observed in several starch-based blends with various polyesters [35,36]. Multilayer films of starch and PLA have higher oxygen and moisture barrier compared to neat PLA [37,38,39]. There are no reports on the compatibilized blends of maleated thermoplastic starch and PLA and their effect on the properties like crystallinity, crystallization rate, barrier, thermal, mechanical, and biodegradability.

In this paper, we report on using inexpensive, REX modified thermoplastic starch particles in the PLA matrix to increase the rate of crystallization and percent crystallinity of PLA. The MTPS-filled PLA polymer films were found to improve oxygen and water vapor permeability without any effect on biodegradability. Crystallinity, crystallization kinetics, and barrier properties were studied and compared with neat PLA. Mechanical and thermal properties as well as morphology of the MTPS-filled thermoplastic PLA were also analyzed. This MTPS could be used as bio-based and biodegradable nucleating agent with a responsible end of life option and a replacement for talc of inorganic origin.

## 2. Materials and Methods

### 2.1. Materials

High amylose corn starch with an initial moisture content of 12.8% (*w/w*) was obtained from National Starch (Bridgewater, NJ, USA). Glycerol was obtained from J.T. Baker (Phillipsburg, NJ, USA) and was used as received. 2,5-bis(tert-butyl-2,5-dimethylhexane), 90% (Luperox 101), and Maleic anhydride (MA) were obtained from Sigma–Aldrich (Milwaukee, WI, USA). Ingeo^TM^ biopolymer 3001D, a commercially available semi-crystalline grade of polylactide (PLA) was supplied from NatureWorks LLC (Minnetonka, MN, USA). It had a molecular weight Mw of 128,000 Da and polydispersity of 1.52. It was prepared from the polymerization of L-lactide and had a meso content of 9%.

### 2.2. Preparation of Maleated Thermoplastic Starch (MTPS) and Polylactide (PLA)/MTPS Blends

**Figure 2:**
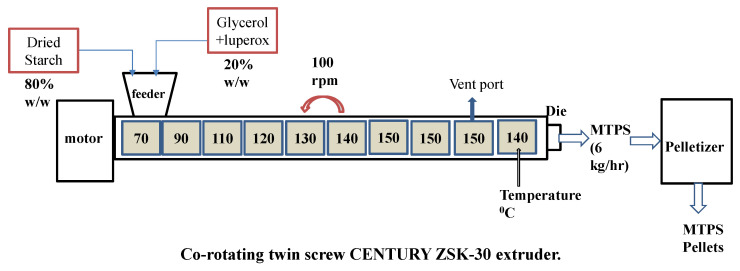
Preparation of maleated thermoplastic starch (MTPS) using reactive extrusion.

MTPS was prepared in a co-rotating twin-screw CENTURY ZSK-30 extruder (MI, USA). The screw diameter and transport length were 30 mm and 1260 mm respectively with L/D ratio of 42. Inherent moisture of starch is reduced before the reactive extrusion because it can interfere with the reactivity of glycerol and can cause foaming of the extrudate. Therefore, the corn starch was dried for 48 h in the oven at a temperature of 65 °C to reduce its moisture content below 1%. MA was used as a promoter for enhanced grafting of glycerol on starch. The details for the reaction chemistry can be found in previous work by Raquez et al. [23,24]. MA (2% by wt.) was ground to fine the powder using a mortar and pestle and was premixed with dry starch (800 g). Luperox (1.1 g) was mixed with glycerol (200 g) and the mixture was then fed into the extruder directly via a peristaltic pump. The feeder was calibrated to get the ratio of 80:20 (starch: glycerol) [16]. The temperature profile was set as 70/90/110/120/130/140/150/150/150/140 °C from the feed port to the die. The screw speed was set at 100 rpm and the melt temperature was 150 °C. The vent port was kept open to remove any moisture and water formed during the reaction. The extrudate coming out of the extruder was air-cooled and pelletized simultaneously using Scheer Bay pelletizer as shown in Figure 2.

Both polylactide and MTPS quickly absorb moisture from the atmosphere. Therefore, they were dried at 55 °C for 12 h before reactive extrusion. Then, MTPS and PLA pellets were mixed in various proportions of 1–10 wt. % in an aluminum tray before feeding. The detailed compositions are listed in the table below (Table 2).

The temperature profile used on the extruder going from the feed section to the die is as follows: 150/160/165/170/180/180/175/175/160/155 °C. These temperatures were selected based on the processing temperatures required for semicrystalline PLA. The screw speed and throughput were 100 rpm and 130 g/min. The extrudate was quenched in a water bath and was then pelletized. The resulting pellets were dried overnight in an oven at 50 °C and then stored in vacuum-sealed bags before using for any further characterization.

### 2.3. Soxhlet Extraction

Selective solubility of glycerol in acetone was used to establish and determine percent covalent grafting of glycerol [23,24]. The MTPS pellets prepared were ground to a fine powder and about 5 g of sample was put in a pre-dried and pre-weighed cellulose extraction thimble. The thimbles were then inserted in the soxhlet extractor connected to a 500 mL round bottom flask containing around 200–250 mL acetone. The flasks were heated, and the solvent was allowed to reflux. The extraction was continued for 72 h. After the extraction, the thimbles were removed; residue and extract were separated and dried overnight at 70 °C. The dried thimble with residue was weighed again and the weight change in the residue was calculated. The reproducibility of the results was confirmed by testing three replicates for each sample. It was expected that the covalently grafted glycerol will not get extracted in acetone and there will be a weight gain in the residue. Percent grafting was calculated from the mass balance as shown in Equation (1).

(1) $$\%\;grafting=\frac{\left|W_{1}-W_{2}\right|}{W_{1}}\times 100$$

where, *W*_1_ is the weight of glycerol present in the sample originally and *W*_2_ is the glycerol in the extract after 72 h. i.e., free glycerol.

### 2.4. Thermal Analysis

The degradation temperature of samples was obtained by thermogravimetric analysis (TGA). TGA measurements of all the samples were conducted under an inert atmosphere of nitrogen using a TGA Q50 (TA Instruments, New Castle, DE, USA). The general sample weight used was 5–7 mg. The sample was placed in an aluminum pan and was heated to 600 °C at the rate of 10 °C/min. The weight loss (%) of a sample as a function of temperature (°C) was obtained from this analysis. Also, the thermal transitions of the samples were obtained by using a differential scanning calorimeter (DSC). The sample was heated to 200 °C in DSC Q20 (TA Instruments, New Castle, DE, USA) with a heating rate of 10 °C/min and held for 5 min to erase thermal history. The sample was then cooled back to 20 °C and heated again to 200 °C with a heating rate of 10 °C/min. The glass transition temperature (T_g_), melting temperature (T_m_), the crystallinity of samples (%X_c_), enthalpy of melting (Δ*H_m_*), and enthalpy of cold crystallization (Δ*H_c_*) were calculated using TA universal analysis 2000 software. The % crystallinity of PLA samples was calculated from the formula given by Bher et al., 2017 [40,41].

(2) $$Crystallinity\;\left(\%\right)=\frac{\Delta H_{m}-\Delta H_{m}}{\Delta H_{o}\left(1-\alpha \right)}\times 100$$

where, Δ*H_m_* and Δ*H_c_* are enthalpies of melting and crystallization respectively. α is the weight fraction of MTPS in the blends and Δ*H_o_* is the enthalpy of melting for 100% crystalline PLA which was obtained from the literature as 93.1 J/g [29,33,41].

### 2.5. Isothermal Crystallization Analysis

To study the isothermal crystallization kinetics, the samples were heated to 200 °C and maintained for 5 min at that temperature to remove any thermal history. Then they were cooled to the desired crystallization temperatures (90, 95, 100, 105 and 110 °C) at a rate of 20 °C/min and held at that temperature till crystallization was complete, then heated again to 200 °C to obtain the melt temperature and final crystallinity after annealing.

### 2.6. Polarized Optical Microscopy (POM)

POM observation was performed on an Olympus BH-2 microscope (Olympus corp., Tokyo, Japan) with crossed-polarizers, equipped with a digital camera system and a Mettler Toledo FP82 (Columbus, OH, USA) hot stage. All the samples were first inserted between two microscope coverslips and squeezed at 200 °C to obtain a thin slice. The films were held at 200 °C for 2 min to achieve thermal equilibrium. This was followed by rapid cooling to the selected crystallization temperature of 105 °C. The polarized optical micrographs during isothermal crystallization were recorded after every 90 s to monitor the formation and growth of crystallites.

### 2.7. Mechanical Properties

The injection molded test bars were prepared using a tabletop DSM 15 cc mini extruder (DSM Research B. V., Sittard-Geleen, The Netherlands) and 3.5 cc mini-injection molder (DACA Instruments, Santa Barbara, CA, USA). The injection pressure was set as 140 psi and the cylinder and mold temperatures were 200 and 65 °C, respectively. The samples were stored for 2 days at 25 °C in a humidity chamber with RH of 50% before any analysis. Tensile testing was performed using an Instron model 5565-P6021 (Instron, Norwood, MA, USA) with a 5 kN load cell and grip separation speed of 12.5 mm/min as per ASTM D882. Data from five samples of each formulation were averaged and compared with the properties of neat PLA.

### 2.8. Scanning Electron Microscopy

A JOEL 6610 LV scanning electron microscope (JEOL Ltd., Tokyo, Japan) was used to study the dispersion of MTPS in PLA using the tensile fracture surfaces of all samples. The tensile bars were immersed in liquid nitrogen for ~2 min and then fractured. Fracture surfaces were mounted on aluminum stubs using high vacuum carbon tabs and coated with gold using a sputter coater. A different set of bar specimens were also treated with 6 N HCl for 12 h to remove the MTPS phase from the samples and air-dried for 12 h in a fume hood. Then they were mounted on aluminum stubs as explained before and examined using JOEL at 500× magnification at 10 kV.

### 2.9. Barrier Properties

The barrier properties were measured using Mocon instruments (OX-TRAN Model 2/21 and PERMATRAN-W Model 3/33, Lyons, CO, USA). All the measurements were undertaken at 50% RH for oxygen and 100% for water vapor. Circular films of 3.14 cm^2^ area were used. The thickness of the samples was measured using a micrometer (TMI) and was used to calculate the permeability to oxygen and moisture. Water vapor permeability (*WVP*) is given as:

(3) $$WVP=\frac{WVTR\;}{\Delta P}\times Th$$

Oxygen permeability (*OP*) was calculated from oxygen transmission rate (*OTR*) data using Equation (4):

(4) $$OP=\frac{OTR\;}{\Delta P}\times Th$$

where, *WVTR* is water vapor transmission rate, *OTR* is oxygen transmission rate, *Th* (m) was the thickness of the sample and Δ*P* was the pressure difference between both sides of the sample (Pa) [42].

### 2.10. Aqueous Biodegradability Testing

The biodegradability of neat PLA and PLA + 5% MTPS samples was tested in an aqueous environment. All the tests were performed in an aerobic environment at 30 °C. A respirometric mineralization test system for calculating CO_2_ evolution was set up based on International Standard ISO 14852. The system comprised blank, positive reference (cellulose) and the test material (PLA and PLA + 5%MTPS) for all the runs. All the samples, blanks, and references were run in duplicates. An optimized test medium containing all the nutrients and buffers was prepared according to the ISO standard. Table 3 gives the detailed composition of the mineral solution prepared for all the tests.

Wastewater inoculum was added to all the flasks to obtain the concentration of 5% *v/v* in the test medium as described in ISO 14852. Then the polymer samples were added to these flasks, and they were subjected to the test conditions. A solution of 1 N NaOH was used for trapping the CO_2_ generated from test flasks. CO_2_ trapping is a two-step process as shown below:

NaOH + CO_2_ → NaHCO_3_

 NaHCO_3_ + NaOH → Na_2_CO_3_

1 g of sample was taken from each of the 50 mL NaOH trapping solution and titrated with 0.1 N standardized hydrochloric acid (HCl) solution to find the amount of CO_2_ trapped. The reactions are as follows:

1st end point:   NaOH + HCl → NaCl + H_2_O

            Na_2_CO_3_ + HCl → NaHCO_3_ + NaCl

    2nd end point:   NaHCO_3_ + HCl → NaCl + H_2_O + CO_2_

The titrations were done with the help of an auto titrator to get the volumes of HCl, *V*_1_ and *V*_2_ required for reactions 1 and 2 respectively. The amount of HCl consumed can be used to calculate the *mmoles of CO*_2_ evolved using the following Equation:

(5) $$mmoles\;of\;CO_{2}=\frac{\left(V_{2}-V_{1}\right)*N_{Hcl}*V_{NaOH}}{Wt\;of\;sample}$$

The percentage biodegradation (*%B*) was further calculated by the following Equation:

(6) $$\%\;B=\;\;\frac{\sum {\left(CO_{2}\right)}_{sample}-\sum {\left(CO_{2}\right)}_{blank}}{ThCO_{2}}\times 100$$

$\sum {\left(CO_{2}\right)}_{sample}$ is the amount of carbon dioxide that evolved in a test flask between the start of the test and time *t*; $\sum {\left(CO_{2}\right)}_{blank}$ is the amount of carbon dioxide that evolved in a blank flask between the start of the test and time *t*; *ThCO_2_* is the theoretical amount of carbon dioxide that evolved from the test material. All the values were expressed as *mmoles of CO_2_*. The samples were replaced every 2–3 days in the starting phase when the rate of biodegradation was expected to be maximum and weekly or biweekly in the end [43,44]. Plots of cumulative CO_2_ evolution for all the samples and blanks and % biodegradation vs. time were made for all the samples and compared for any differences between PLA and PLA + 5% MTPS.

## 3. Results and Discussion

MTPS and all the PLA/MTPS blends were prepared in a Century ZSK-30 twin screw extruder (Century Extruders, Traverse City, MI, USA). MTPS was first characterized to ensure sufficient grafting of glycerol and then it was used for blending with PLA.

### 3.1. Percent Grafting for MTPS

**Figure 3:**
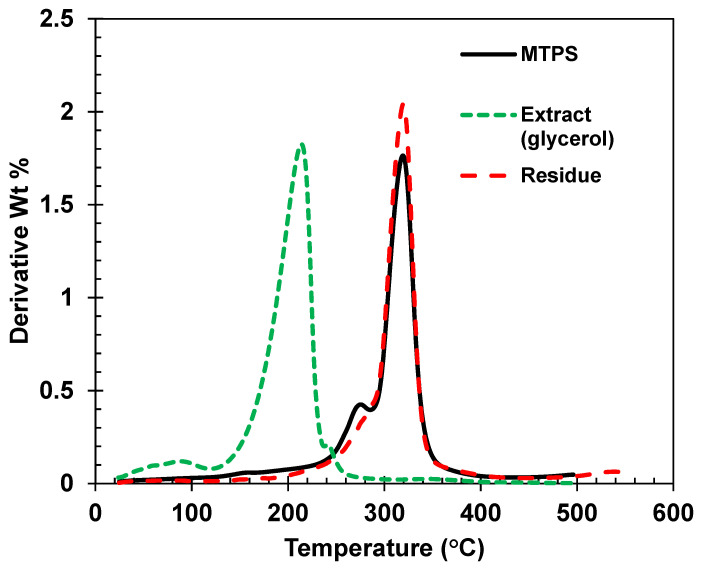
Derivative thermogravimetric (DTG) curves of MTPS, glycerol, and residue showing only MTPS after soxhlet extraction.

Soxhlet analysis provided percent covalent grafting of glycerol to the starch backbone. Acetone was used as the extraction solvent. In this, the covalently bonded glycerol is not extracted, and only free, ungrafted glycerol is extracted by acetone. The results are shown in Table 4. The removal of free glycerol by acetone was further confirmed from the results of TGA analysis as shown in Figure 3. A decrease in the peak corresponding to glycerol was observed in the residue after Soxhlet extraction whereas the TGA of the extract showed a peak only for glycerol with no MTPS indicating that acetone extracts only free glycerol and no MTPS. Results indicated that 79% of added glycerol was chemically grafted on the starch backbone. Similar results have been observed in the previous studies from our group [23,24].

Soxhlet extraction was carried out for the blends of PLA and MTPS as well using dichloromethane as extraction solvent. It was found that the weights of residues and extracts did not change after the extraction. Entire PLA was extracted in the solvent and MTPS remained in the residue. There were no additional peaks in residue or extract TGA which could represent any sign of reaction between PLA and MTPS. Hence, it was concluded that there was no reaction between MTPS and PLA.

### 3.2. Mechanical Testing and Phase Morphology

**Figure 4:**
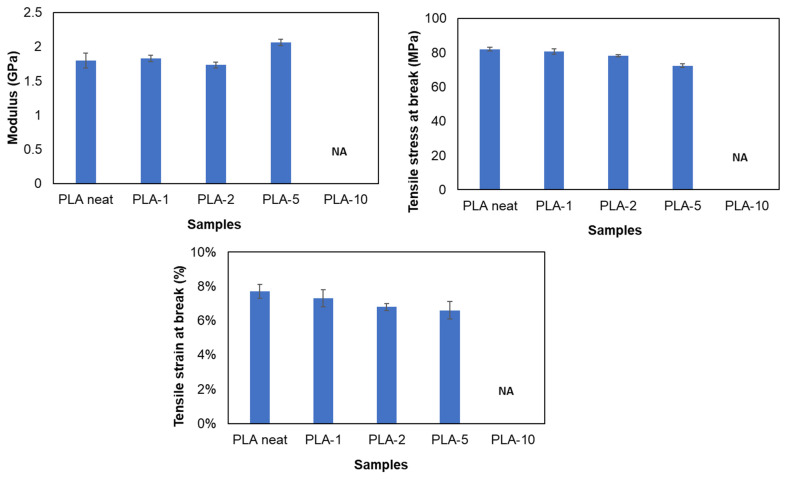
Modulus, tensile stress, and tensile strain graphs for PLA and blends.

**Figure 5:**
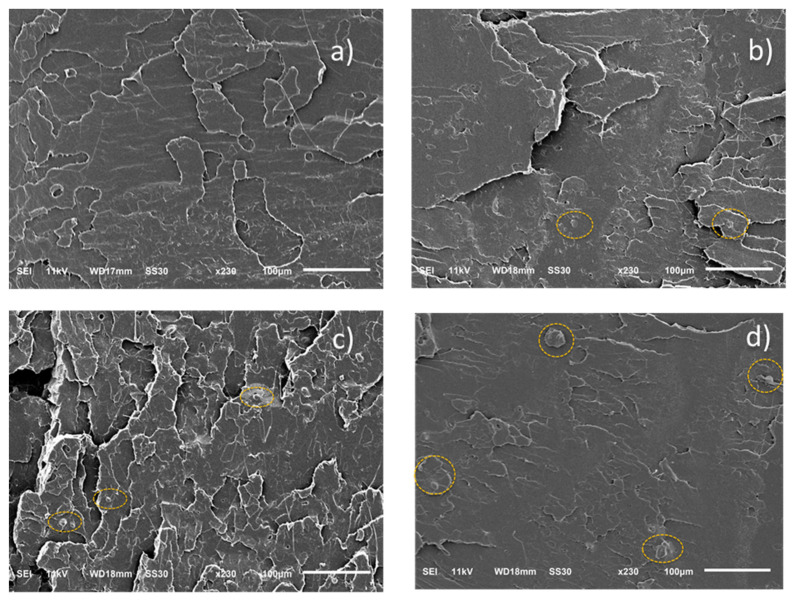
Scanning electron microscopy (SEM) images of fractured tensile surfaces of (**a**) PLA, (**b**) PLA + 1% MTPS, (**c**) PLA + 2% MTPS, (**d**) PLA + 5% MTPS.

**Figure 6:**
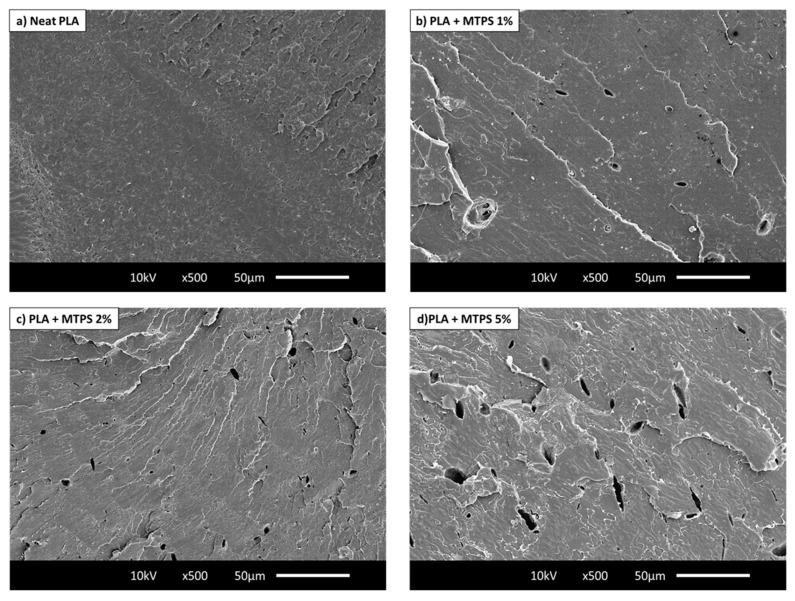
SEM images of the blends after selective extraction of MTPS phase (**a**) PLA, (**b**) PLA + 1% MTPS, (**c**) PLA + 2% MTPS, (**d**) PLA + 5% MTPS.

Figure 4 illustrates the modulus, tensile stress, and strain for neat PLA and the various blends. Table 5 summarizes all the data of tensile stress at yield, tensile stress at break, modulus, and strain at break with averages and standard deviations. The analysis of the tensile testing of neat PLA samples reveals a characteristic brittle behavior of PLA with tensile strength values of ~82 MPa and elongation at break ~8%. The results indicated that there was no significant change in the modulus, tensile stress, and strain after addition of 1% and 2% MTPS (*p* > 0.05). Only for 5% MTPS containing blends, the modulus increased by 15% indicating they were stiffer than neat PLA whereas tensile stress reduced by 12%. Increasing MTPS content in PLA increased the brittleness of PLA bars. Similar results were observed by Wootthikanokkhan 2012 et al. [45], where the modulus of the samples was found to increase with an increasing percentage of starch at the expense of elongation and tensile toughness. For PLA + 10% MTPS, the samples became brittle and broke after loading on the tensile machine. Hence, the readings for modulus, stress, and strain were not recorded. Because of the reduction in mechanical properties, they were not considered for any further analysis. This reduction in mechanical properties could be due to the reduced compatibilization and increased particle size of the MTPS particles in the PLA matrix. This was observed in the SEM images of the samples as explained in Figure 5 and Figure 6.

The morphological behavior of neat PLA and the blends was analyzed using SEM images of tensile fracture surfaces. The main results are reported in Figure 5. The tensile fracture surface of neat PLA shows a smooth and featureless surface which is an indicator of typical brittle behavior. Figure 5b–d show presence of small spherical MTPS particles (shown with dotted yellow circles) with good interfacial adhesion with PLA. They showed a smooth surface and were well wetted by the PLA. The size of MTPS particles seemed to increase with increasing content of MTPS (Figure 5b,d). However, the number of MTPS particles did not increase much and their effect on thermal properties was also negligible.

Figure 6 shows the morphology of the PLA matrix and the PLA/MTPS blends produced by extrusion after selective removal of the MTPS phase. Cavities represent the spaces occupied by MTPS particles before their selective removal by dissolution in concentrated HCl. The proportion of cavities was well correlated with the percentage of MTPS added in the particular blend. Figure 6b–d all show micro size distribution of MTPS particles in the PLA matrix. T of the cavities seemed to increase with increase of MTPS content which suggested a reduction in compatibilization of MTPS. The particle size for MTPS increased from 4.1 ± 1.5 μm for 1% MTPS to 9.5 ± 3.5 μm for 5% MTPS blends. An increase in the domain size is indicative of less compatibilization between PLA and MTPS. This could explain the reduction in mechanical properties for 5% and 10% MTPS containing blends. The 1% and 2% MTPS (Figure 6a,b) had smaller size and more spherical particles which indicated greater compatibilization, and hence no reduction in tensile strength was observed compared to neat PLA. These particle sizes of 4–9 μm were much smaller as compared to the 30 μm size observed by Clasen SH et al. (2015) in the PLA-TPS blends without any compatibilizer.

### 3.3. Thermal Analysis

**Figure 7:**
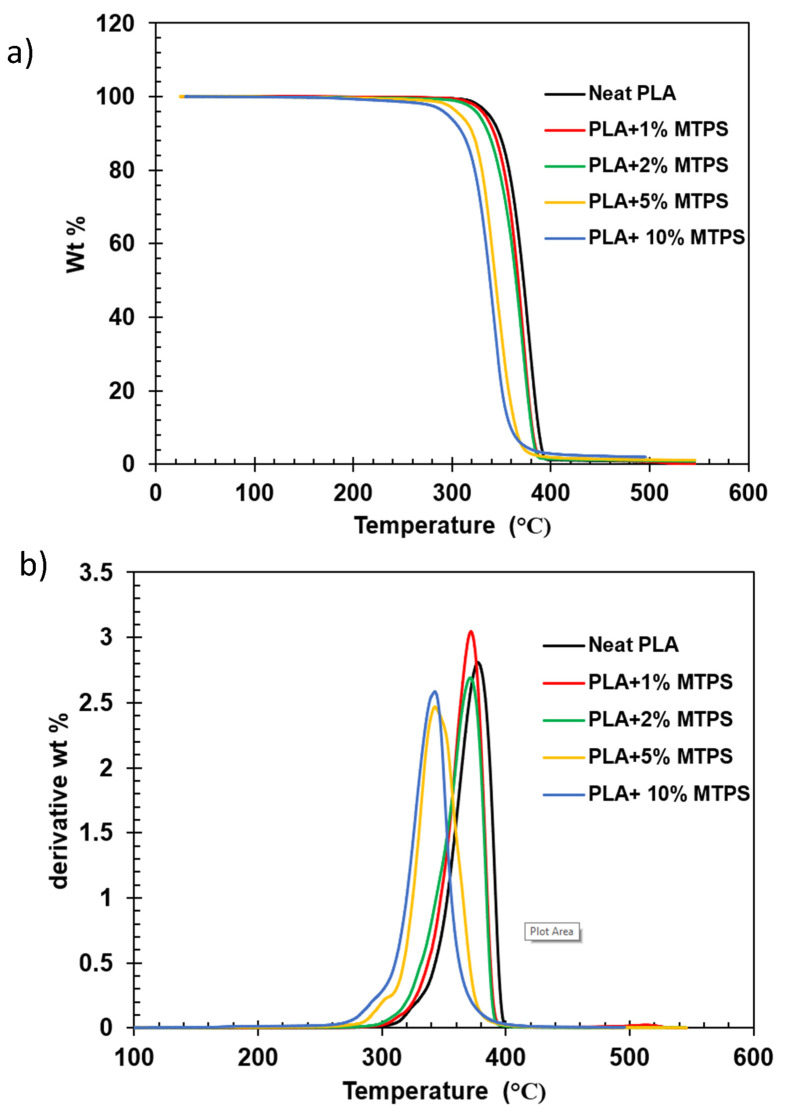
(**a**) TGA and(**b**) DTG curves for PLA and PLA/MTPS blends.

Thermogravimetric (TGA) and derivative (DTG) graphs of the blends containing 1%, 2%, 5%, and 10% MTPS along with neat PLA are shown in Figure 7. With increment in MTPS until 5%, the weight loss in the first part of the curve (<200 °C) was almost the same and was found to be less than 0.5% which is a significant feature since it represents the range and possible process temperatures for the blends after production. With 10% MTPS, the weight loss until 200 °C increased to 0.7%. Thermal stabilities of the blends were also characterized by the temperatures at which 5% (T_5%_), 10% (T_10%_), and peak wt. loss (T_peak_) occurred. Increasing the percentage of MTPS caused a steady decrease in 5% and 10% wt. loss temperatures. The peak degradation temperatures shifted towards the lower temperature with increase in the percentage of MTPS. The results are summarized in Table 6.

**Figure 8:**
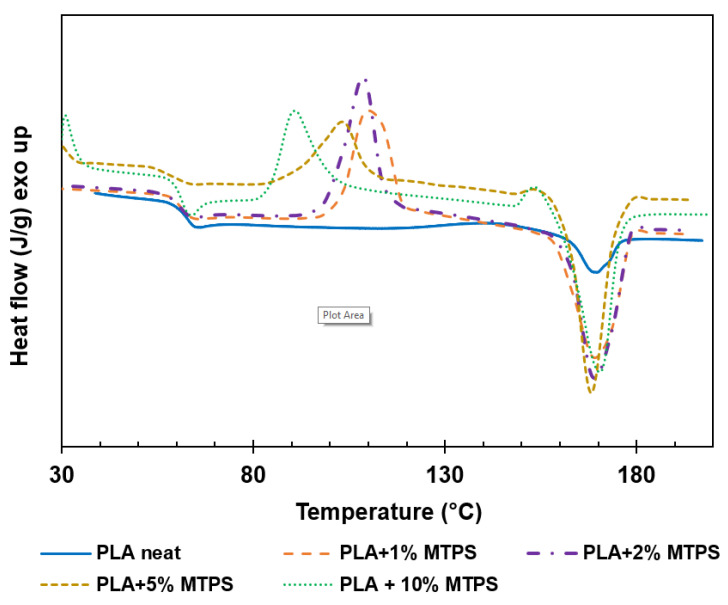
Differential scanning calorimetry (DSC) thermograms of PLA and the blends.

The glass transition temperature T_g_ (°C), crystallization temperature T_c_ (°C), melting temperature T_m_ (°C), enthalpy of crystallization (J/g), enthalpy of melting (J/g), and crystallinity (%) data of PLA and modified PLA pellets were determined from the DSC analysis and are given in Table 7. All the thermal properties were obtained from the second heating curve (Figure 8). Thermal degradation and mechanical properties of PLA with 10% MTPS (explained in Section 3.4) were significantly lower as compared to neat PLA. Hence, that sample was not used for further testing of isothermal crystallization, barrier properties, etc. Figure 8 shows the second heating curves for PLA and PLA/MTPS blends. T_g_ was found to decrease negligibly with addition of MTPS to PLA whereas T_c_ reduced from 113.8 to 103.1 °C. In our opinion, this decrease might be due to the migration of some glycerol from the MTPS phase to the PLA phase. This might lead to the formation of plasticized PLA with lower T_g_.

**Figure 9:**
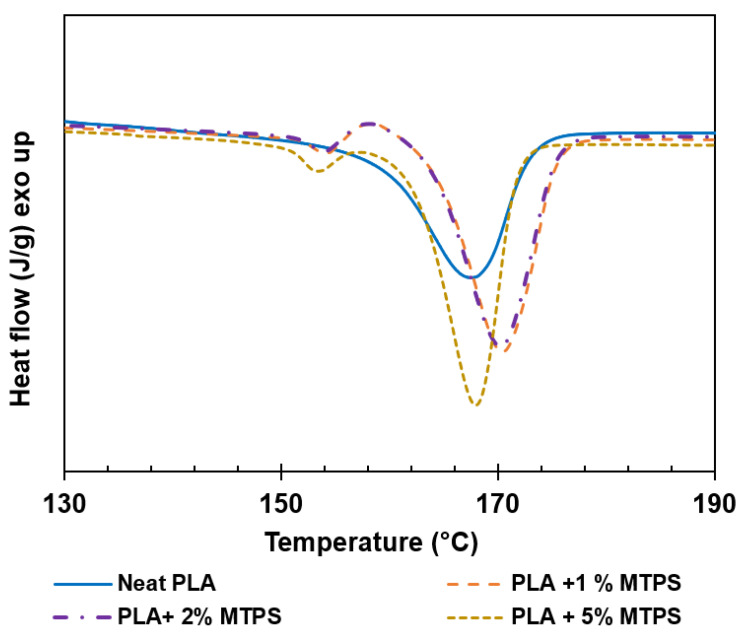
Melting curves for PLA blends after annealing.

From DSC of neat PLA, it seemed that there was just a little endothermic peak on the curve of pure PLA and the crystallinity was about 7.74%. After adding MTPS, an exothermic peak appeared on the heating curve. In the course of heating, more crystals were formed and hence the crystallinity of PLA increased with increasing concentration of MTPS suggesting its function as a nucleating agent similar to starch [32]. The final crystallinity of the samples was also calculated after annealing of the samples as explained in the isothermal crystallization analysis. Figure 9 shows the melting curves for annealed samples. The final crystallinity of the samples after annealing increased to 50.6% with 5% MTPS.

### 3.4. Isothermal Crystallization Kinetics

**Figure 10:**
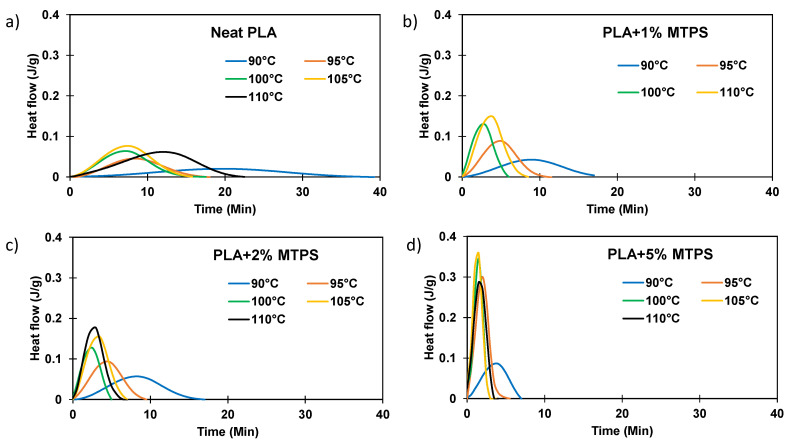
DSC melting thermograms of (**a**) PLA and blends (**b**) PLA + 1%MTPS, (**c**) PLA + 2%MTPS, (**d**) PLA + 5% MTPS at various temperatures.

The isothermal crystallization isotherms of PLA and the blends obtained by cooling the molten polymer to the selected crystallization temperature (T_c_) are as shown in Figure 10. The shape of the exotherm was dependent on T_c_. The time required for crystallization was found to be minimum at 100 °C. Above and below that temperature, the isotherm became flatter, and the time required for complete crystallization increased. A similar effect was observed for the PLA/MTPS blends as well. Fractional crystallinity *X_t_* vs. time is the ratio of the area of the endotherm until time *t* divided by the total area of the endotherm, as shown in Equation (3).

(7) $$X_{t}=\frac{X_{c}\left(t\right)}{X_{c}\left(t_{\infty }\right)}=\frac{\int \int_{0}^{t}\frac{dHc\left(t\right)}{dt}dt}{\int_{0}^{\infty }\frac{dHc\left(t\right)}{dt}dt}\;$$

where *H_c_* is the heat flow at time *t*, and $t_{\infty }$ is the end time for complete crystallization.

**Figure 11:**
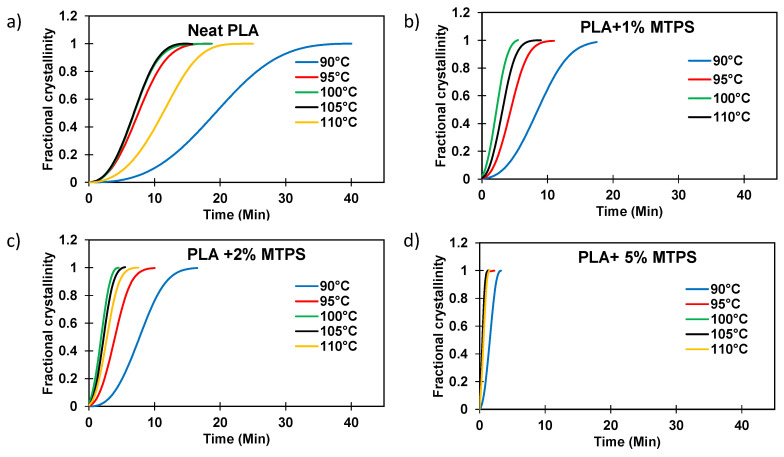
Fractional crystallinity vs. time of (**a**) PLA and blends (**b**) PLA + 1%MTPS, (**c**) PLA + 2%MTPS, (**d**) PLA + 5% MTPS at various temperatures.

Typical crystallization isotherms showing the degree of crystallinity *X(t)* vs. time were plotted for PLA and the blends as shown in Figure 11. It was observed that the rate of crystallization increased with an increasing amount of MTPS addition and was fastest for neat PLA as well as all the blends for the temperature of 100 °C.

The Avrami Equation (4) was used for studying the isothermal crystallization behavior of PLA and the blends. It is often written in logarithmic form as shown in Equation (5).

(8) $$X\left(t\right)=1-\exp\left(-kt^{n}\right)\;$$

(9) or ln[−ln(1−X(t)]=nlnt+lnk

where *X(t)* is the fractional crystallinity at time *t*, *k* is the overall kinetic rate constant, and *n* is the Avrami exponent, which depends on the mechanism of nucleation and the form of crystal growth. The rate constant *k* contains the nucleation and growth parameters for crystallization.

**Figure 12:**
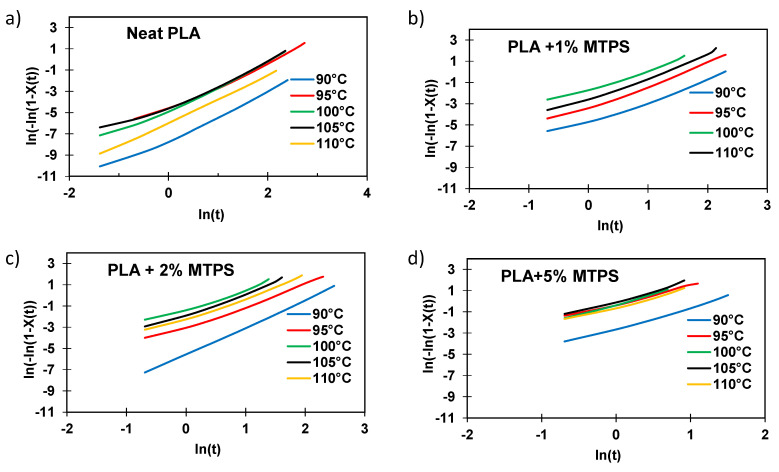
Plots of ln (ln(1 − X(t)) vs. ln t of (**a**)PLA and blends (**b**) PLA+1%MTPS, (**c**) PLA + 2%MTPS, (**d**) PLA + 5% MTPS at various temperatures.

It was observed that the total crystallization time was reduced significantly (<8 min for 5% MTPS) by the addition of MTPS as compared to neat PLA (~20 min at 100 °C). The half time (*t_1/2_*) was calculated and reported for all the temperatures in Table 7. Avrami plots of heat flow vs. time and *ln[−ln(1−X(t))]* versus *ln(t)* were plotted to obtain the values of *k* and *n* as shown in Figure 11 and Figure 12 respectively. The crystallization rates (*k*) were much higher for the blends containing MTPS as compared to neat PLA at all temperatures indicating that MTPS increased the crystallization rate of PLA. Also, from Avrami analysis results as shown in Table 7, two-dimensional crystal growth was observed as *n* values were around 2 [5]. Thus, it can be concluded that MTPS was acting as a nucleating agent for PLA. The half crystallization time, *t_1/2_*, the time in which 50% of the total crystallinity is achieved, was calculated using Equation (6).

(10) $$t_{1/2}=\ln{\left(\frac{2}{k}\right)}^{1/n}$$

**Figure 13:**
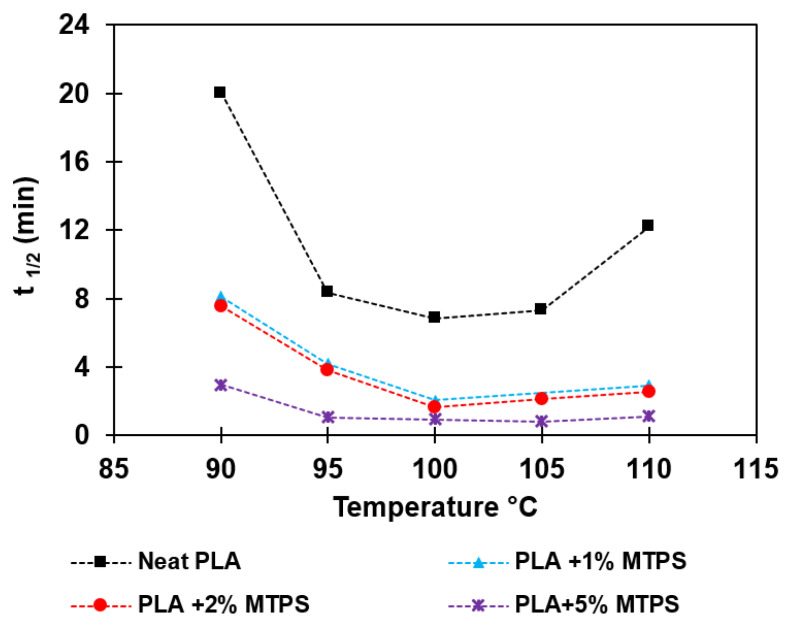
Half time for crystallization vs. isothermal crystallization temperatures for PLA and blends.

Saddle-shaped curves were obtained by plotting Tc vs. *t_1/2_* for PLA and its blends with MTPS as shown in Figure 13. As the MTPS content increased, *t_1/2_* values decreased and the rates for crystallization k were found to increase. The minimum *t_1/2_* for neat PLA was observed as 6.83 min at 100 °C and 1.66 and 0.94 min for 2% and 5% of MTPS at 100 °C.

**Figure 14:**
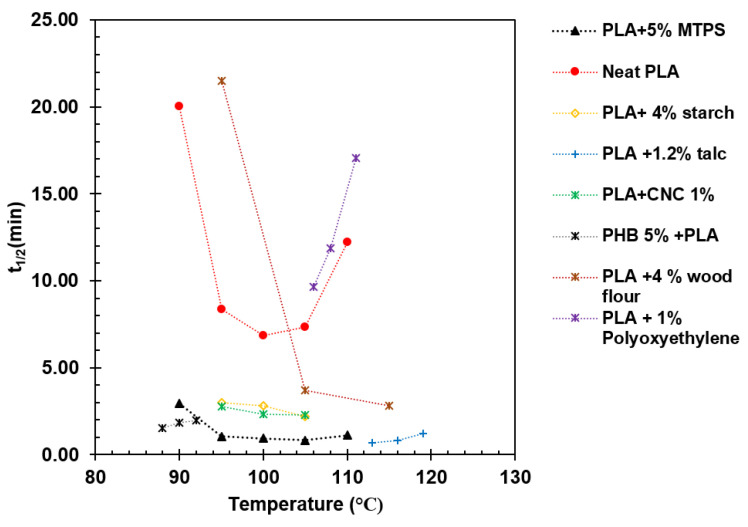
Comparing the t_1/2_ values of PLA/MTPS blends with other nucleating agents.

The values for t_1/2_ obtained for PLA/MTPS blends were compared with several nucleating agents including starch, talc, CNC, wood flour, polyoxymethylene, etc. [5,7,8,9,11,12,33,46,47] from the literature. Figure 14 shows the comparison for t_1/2_ values of other nucleating agents compared to MTPS. The t_1/2_ values for MTPS were found to be lower compared to all the other nucleating agents except talc. Talc is one of the most effective nucleating agents for PLA. 1–2% of talc is commonly added to decrease the t_1/2_ of PLA to less than one minute [33]. Thus, MTPS, although not as effective as talc, could be a completely bio-based and biodegradable nucleating agent as opposed to talc of inorganic origin.

### 3.5. POM Analysis

**Figure 15:**
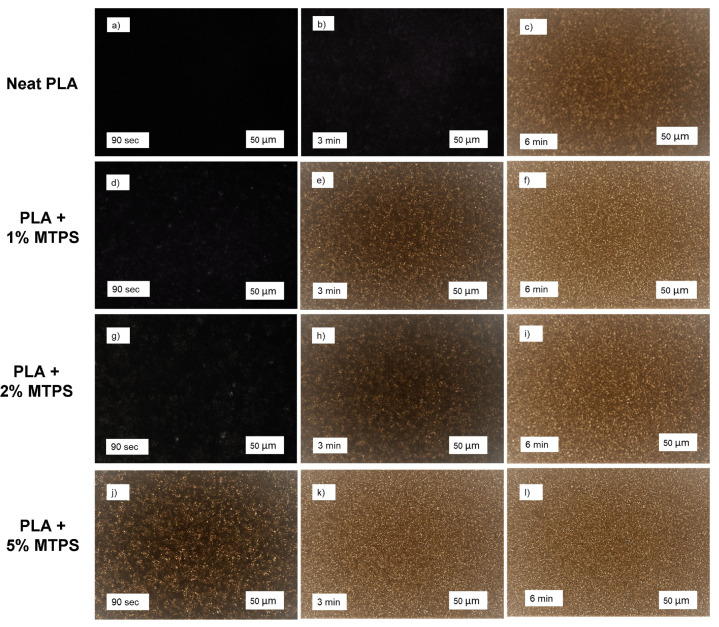
Polarized optical microscopy (POM) images of PLA/MTPS blends with MTPS content of 0%: (**a**–**c**); 1%: (**d**–**f**); 2%: (**g**–**i**); 5%: (**j**–**l**).

The effect of MTPS on crystal morphology and size was studied using polarized optical microscopy. Figure 15 shows the morphology of crystals for all the compositions after crystallization at 105 °C for 6 min. As expected, neat PLA showed larger size spherulites and the rate of formation of spherulites was less as compared to other samples. As shown in Figure 15b, neat PLA started forming the spherulites well after holding it at 105 °C for 3 min, whereas, for all other PLA/MTPS samples we could see a good number of spherulites by that time (Figure 15e,h,k). For PLA + 5% MTPS, this was even faster, and the crystals were visible within 90 s (Figure 15j). This agreed well with the k values obtained by Avrami analysis for the samples which indicated 98-fold faster crystallization for PLA + 5% MTPS at 100 °C (Table 8). This can be attributed to the nucleation effect of MTPS, which provides much more heterogeneous nuclei, reduces the spherulite size, and speeds up the crystallization process. 

### 3.6. Permeability Studies

**Figure 16:**
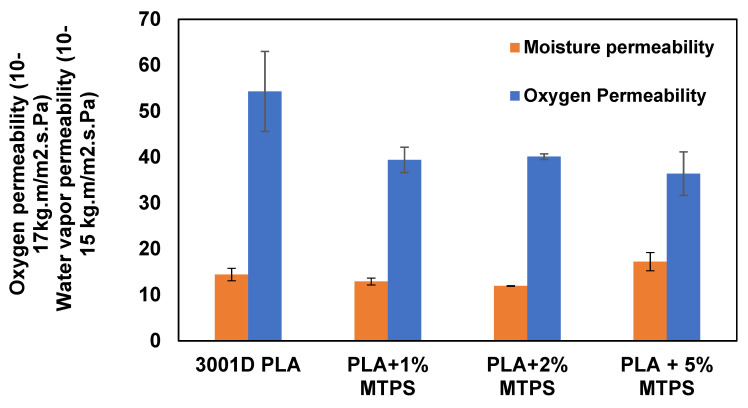
Effect of MTPS content on moisture permeability (WVP) and oxygen permeability (OP) of PLA films.

Starch based films have demonstrated their good oxygen barrier properties in previous studies [13,37,38,39,42]. Figure 16 summarizes the effect of MTPS on WVP and OP of PLA films. A decrease of 33% and 27% in oxygen permeability was observed by adding 5% and 1% MTPS, respectively. This improvement can be attributed to increased crystallinity. Crystalline regions in PLA form the impermeable regions which create a tortuous path for the diffusion for permeants, which leads to lower permeability [48,49,50,51,52]. Also, high oxygen barrier properties of starch might also be helpful in reducing the OP of the films with blends. WVP and OP show a significant reduction for the addition of 1% MTPS, whereas it becomes less significant as the concentration is increased to 2% and 5%. (Figure 16). This could also be explained by the particle size of MTPS observed from the SEM analysis. The 1% MTPS blends had an average particle size of 4.1 ± 1.5 μm whereas, for 5% MTPS, the size was almost double: 9.5 ± 3.5 um. More small particles could have caused a greater number of tortuous paths leading to reduced permeability. The water vapor permeabilities of the blends did not show any significant increase even after addition of hydrophilic MTPS. This might be due to the morphology of the blend in which the MTPS particles were observed to be surrounded by hydrophobic PLA matrix thus shielding it from water (Figure 5 and Figure 6).

### 3.7. Biodegradability Studies

**Figure 17:**
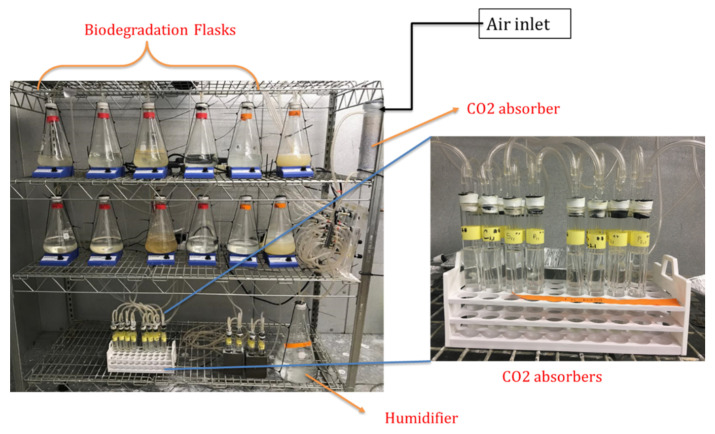
Experimental aqueous biodegradation setup.

Figure 17 shows the test setup for aqueous biodegradation according to ISO 14852. The system was kept in a dark, temperature-controlled room maintained at 30 °C. The test flasks were agitated throughout the run with the help of magnetic stirrers. Air inlet was passed through NaOH solution to get CO_2_-free air. This air was then divided and passed through flowmeters for each bioreactor at a constant flow rate. The CO_2_ evolved from the flasks was collected in NaOH solution and titrated with HCl to determine the CO_2_ that evolved from the samples and % biodegradation as described in Section 2.9.

**Figure 18:**
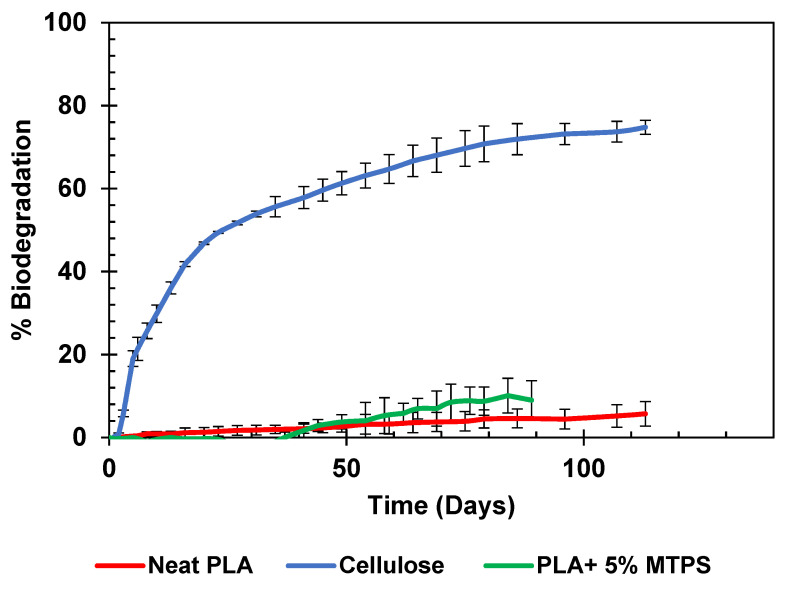
Aqueous biodegradation curves for PLA and PLA + 5% MTPS.

The average % biodegradation curves for cellulose and PLA and PLA + 5% MTPS at 30 °C are as shown in Figure 18.

It was observed that neat PLA and PLA + 5% MTPS have almost the same biodegradation curve. Both showed negligible biodegradation in aqueous environment (<10%) at the end of the test. Therefore, it can be concluded though MTPS acted as a nucleating agent and increased the crystallinity and crystallization rate of PLA, it was well embedded in the hydrophobic PLA matrix and hence did not change the biodegradation properties of PLA. Poor biodegradability of PLA was attributable to the temperature at which the aqueous biodegradation test was carried out −30 °C. The glass transition temperature of PLA is 58 °C. Below this temperature, PLA does not biodegrade easily due to the polymer segments behaving as a glass with little or no mobility of the polymer chains. Hence, no difference could be observed at lower temperature studies. Similar to any chemical reaction the rate of biodegradation depends on temperature and is expected to increase as temperature increases. A higher biodegradation rate is expected in the high temperature composting environment testing which is the ideal environment for PLA biodegradation. PLA reaches 80–90% biodegradation within 60–90 days in composting environment at temperatures of 58 °C as observed in several studies [53,54,55]. These compatibilized blends with maleic anhydride are also expected to have higher biodegradability as compared to PLA and pure starch blends in the composting environment [34,56]. During composting, the presence of MA might lead to the formation of an acid group due to its reaction with water. That can accelerate the chain scission in PLA resulting in faster biodegradation [57]. Further testing for biodegradation in composting environment needs to be done to validate this hypothesis.

## 4. Conclusions

Maleated thermoplastic starch (MTPS) was successfully prepared by reacting glycerol with corn starch using maleic anhydride as a promoter. It was found that 79% of added glycerol was grafted on the starch during reactive extrusion. The dual effect of MTPS as a nucleating agent and barrier property enhancer for PLA was studied. MTPS increased the rate of crystallization of PLA significantly (98-fold at 100 °C). A decrease in glass transition and thermal degradation temperature was observed with increasing concentration of MTPS. Percent crystallinity of PLA increased from 7.7% to 28.6% by addition of 5% MTPS whereas total crystallinity of the blends was as high as 50.6% after annealing. SEM images of tensile fractured samples showed good interfacial adhesion and wetting between MTPS and PLA. The 1% MTPS showed the best compatibilization with domain sizes of 4.1 ± 1.5 μm as observed from the SEM images of tensile bars after selective extraction of the MTPS phase. Melt isothermal crystallization kinetics using Avrami analysis showed a drastic reduction in half crystallization time t_1/2_ from 20 min to less than 1 min with addition of 5% MTPS. An increased crystallization rate was also confirmed by POM images of neat PLA and the blends. More number of smaller spherulites were observed with an increasing percentage of MTPS in the blends. MTPS was found to be more effective as compared to many other nucleating agents used for PLA such as starch, CNC, wood flour, polyoxyethylene, etc. Oxygen permeability values of PLA were reduced by 27% by the addition of just 1% MTPS whereas water vapor permeability values remained constant. No significant change in the mechanical properties of the blends was observed as opposed to neat PLA until 5% addition of MTPS by weight. A small decrease in tensile stress and elongation at break was observed after addition of 10% MTPS which could be explained by the brittle nature of MTPS. There was no significant increase in the aqueous biodegradability of these blends compared to neat PLA, which suggested that the MTPS particles were well embedded in the hydrophobic PLA matrix. It is expected that the presence of starch and MA will enhance the biodegradability of these blends in a composting environment. Further studies need to be undertaken to obtain the actual experimental data supporting this hypothesis. These MTPS/PLA blends have demonstrated improved barrier and crystallization properties and similar mechanical, thermal and biodegradation properties to neat PLA. These blends have a potential for reduced cost due to use of an inexpensive, naturally abundant, completely bio-based, and biodegradable nucleating agent and can find applications in several food contact packaging purposes.

## Figures and Tables

**Table 1:** Summary of physico-mechanical properties of packaging polymers.

	PLA	PET	LDPE	PS	PP	Starch	Ref.
**Tensile Strength (MPa)**	50–80	50–70	10–20	40	44	<6	[11,12]
**Glass Transition Temperature (°C)**	60	70	−130	170	100	_	[11]
**Melting Temperature (°C)**	160–170	260	110	160	222	120–150 (T_d_)	[16,17]
Oxygen permeability (cm^3^ × mm/m^2^ × d × atm) *	7.9–52	1.18	98–453	98.5–171	35–377	0.35–2.19	[17,18,19]
Water permeability (cm^3^ × cm/cm^2^ × s × mmHg (×10^10^)) *	139–617	130	68	123–600	35	533–3300	[4,13,14,15,19,20]

* Values from the literature have been converted to the same units for ease of comparison.

**Table 2:** Sample name and composition.

Sample	PLA wt. %	MTPS wt. %
PLA	100	-
PLA-1	99	1
PLA-2	98	2
PLA-5	95	5
PLA-10	90	10

**Table 3:** Mineral solution composition for the test.

1 L Mineral Solution	g
Solution A	
KH_2_PO_4_ (anhydrous)	3.75
Na_2_HPO_4_·2H_2_O	8.73
NH_4_Cl	0.2
Solution B	
MgSO_4_·7H_2_O	2.25
Solution C	
CaCl_2_·2H_2_O	3.64
Solution D	
FeCl_3_·6H_2_O	0.025 g
Wastewater inoculum (mL)	50
Distilled water (mL)	Remaining

**Table 4:** Results for percent grafting of glycerol by Soxhlet extraction.

Sample	1	2	3
Weight (g)	5.009	5.015	5.003
Starch (g)	4.007	4.012	4.002
Glycerol (g)	1.002	1.003	1.001
Extract (g)	0.214	0.221	0.201
Residue (g)	4.794	4.793	4.801
%Grafting	78.58	77.87	79.90
Average Grafting (%)	78.7 ± 0.7		

**Table 5:** Effect of MTPS content on tensile properties of PLA.

Materials	Modulus	Tensile Stress at Yield	Tensile Strain at Break	Tensile Stress at Break
	(MPa)	(MPa)	(mm/mm)	(MPa)
PLA neat	1796.45 ± 110.08 ^a^	82.10 ± 1.14 ^a^	0.077 ± 0.004	73.30 ± 2.74
PLA-1	1825.34 ± 47.76 ^a^	80.67 ± 1.60 ^a^	0.073 ± 0.005	72.76 ± 2.01
PLA-2	1748.27 ± 44.03 ^a^	78.210 ± 0.65 ^a^	0.068 ± 0.002	73.63 ± 2.17
PLA-5	2059.88 ± 47.09 ^b^	72.38 ± 1.14 ^b^	0.066 ± 0.005	67.77 ± 3.97

Different superscript letters within the same column indicate significant differences among formulations.

**Table 6:** Thermogravimetric analysis (TGA) of PLA and blends.

Sample	T_5%_ (°C)	T_10%_ (°C)	T_peak_ (°C)
PLA	337.4	347.7	377.5
PLA-1	333.4	342.7	371.9
PLA-2	327.1	336.9	371.0
PLA-5	308.4	320.9	343.3
PLA-10	295.68	310.7	342.6

**Table 7:** Thermal transition temperatures and percent crystallinity of modified PLA samples.

	T_g_ (°C)	T_m_ (°C)	T_c_ (°C)	ΔH_m_ (J/g)	ΔH_c_ (J/g)	% Crystallinity	% Crystallinity after Annealing
PLA neat	63.30	171.50	113.86	40.94	33.74	7.74	43.18
PLA + 1% MTPS	62.30	171.00	111.40	40.72	31.13	10.42	47.28
PLA + 2% MTPS	61.38	170.57	108.46	45.45	32.55	14.15	48.20
PLA + 5% MTPS	59.50	161.40	103.10	45.03	19.71	28.66	50.61

**Table 8:** Crystallization half times and Avrami constants for PLA samples at different temperatures.

Sample	Temperature (°C)	t_1/2_ (min)	*n*	ln k	k
**Neat PLA**	90	20.03	2.520	−7.919	3.64 × 10^−4^
	95	8.36	1.890	−4.380	1.25 × 10^−2^
	100	6.83	2.320	−4.825	8.03 × 10^−3^
	105	7.32	2.420	−5.184	5.61 × 10^−3^
	110	12.21	2.240	−5.972	2.55 × 10^−3^
**PLA + 1% MTPS**	90	8.12	2.198	−4.969	6.95 × 10^−3^
	95	4.19	2.127	−3.412	3.30 × 10^−2^
	100	2.03	1.797	−1.641	1.94 × 10^−1^
	110	2.91	2.084	−2.593	7.48 × 10^−2^
**PLA + 2% MTPS**	90	7.54	2.619	−5.657	3.49 × 10^−3^
	95	3.83	1.964	−3.004	4.96 × 10^−2^
	100	1.66	1.811	−1.289	2.76 × 10^−1^
	105	2.10	1.946	−1.809	1.64 × 10^−1^
	110	2.55	1.958	−2.200	1.11 × 10^−1^
**PLA + 5% MTPS**	90	2.94	1.960	−2.479	8.38 × 10^−2^
	95	1.06	1.410	−0.447	6.40 × 10^−1^
	100	0.94	2.010	−0.242	7.85 × 10^−1^
	105	0.82	1.780	−0.018	9.82 × 10^−1^
	110	1.12	1.740	−0.566	5.68 × 10^−1^
